# Qualitative study on domestic social robot adoption and associated security concerns among older adults in Slovenia

**DOI:** 10.3389/fpsyg.2024.1343077

**Published:** 2024-01-25

**Authors:** Boštjan Žvanut, Anže Mihelič

**Affiliations:** ^1^Department of Nursing, Faculty of Health Sciences, University of Primorska, Izola, Slovenia; ^2^Faculty of Criminal Justice and Security, University of Maribor, Ljubljana, Slovenia

**Keywords:** older adults, social robots, domestic robots, technology adoption, qualitative research, information security, cyber security

## Abstract

**Introduction:**

Despite the increasing use of domestic social robots by older adults, there remains a significant knowledge gap regarding attitudes, concerns, and potential adoption behavior in this population. This study aims to categorize older adults into distinct technology adoption groups based on their attitudes toward domestic social robots and their behavior in using the existing technology.

**Methods:**

An exploratory qualitative research design was used, involving semi-structured interviews with 24 retired Slovenian older adults aged 65 years or older, conducted between 26 June and 14 September 2023.

**Results:**

Four distinct groups of older adults were identified: (1) Cautious Optimists, (2) Skeptical Traditionalists, (3) Positive Optimists, and (4) Technophiles based on eight characteristics.

**Discussion:**

These groups can be aligned with the categories of the Diffusion of Innovation Theory. Privacy and security concerns, influenced by varying levels of familiarity with the technology, pose barriers to adoption. Perceived utility and ease of use vary considerably between groups, highlighting the importance of taking into account the different older adults. The role of social influence in the adoption process is complex, with some groups being more receptive to external opinions, while others exhibit more autonomous decision-making.

## 1 Introduction

Robotics, as a field, has transcended its traditional industrial roles, becoming increasingly being integrated into various aspects of daily life. The European Commission’s survey shows this integration, revealing that a significant majority (70%) of EU citizens have a positive attitude toward robots. This positive perception is particularly pronounced among those who have direct personal experience with robots. The survey highlights the social consensus on the role of robots, particularly in tasks considered too dangerous or too demanding for humans, such as space exploration, manufacturing, and emergency services. This widespread acceptance underscores a decisive shift in the public perception of robots, which are no longer seen merely as industrial tools but as indispensable helpers in numerous areas (European Commission, 2012).

Distinct from their industrial counterparts, domestic social robots represent a segment of robotics, that is becoming increasingly prevalent in personal spaces such as homes and healthcare facilities. Domestic social robots are autonomous devices designed for household environments to interact and communicate with humans in a socially meaningful way (Fong et al., 2003). These robots are typically equipped with sensors, cameras, microphones, and artificial intelligence. They can perform tasks such as reminding users of appointments, providing companionship, assisting with daily chores, and monitoring health and safety. They are commonly designed to be user-friendly and exhibit human-like characteristics (e.g., eyes) or behaviors (e.g., listening, answering questions) to facilitate natural interaction and acceptance by humans. Advances in machine learning and robotics have been instrumental in making these social robots more practical and affordable for domestic use. Their functions go beyond simply performing tasks and include features such as companionship, interactive games, and multimedia entertainment. This marks a shift toward robots that are not only functional but also emotionally engaging and interactive.

The market acceptance of domestic social robots, however, remains a multifaceted challenge. As studies (e.g., Bartneck et al., 2005; Wu et al., 2014; David et al., 2022) have shown, the presence of robots in daily life is no guarantee of their acceptance or the willingness of users to interact with them. This complexity is reflected in the varied market successes of different robots. For example, while robots such as Buddy and Astro have gained a foothold in the market, others such as Pepper and Jibo have faced challenges as low demand has led to production pauses or cancelations (Heater, 2019; Wakefield, 2021). These examples illustrate the nuanced nature of the domestic social robot market, where consumer acceptance depends on a complex interplay of factors, including personal experience, perceived utility, and societal norms.

Comprehensive models have been developed and proposed in the literature to better understand and address these challenges. The Model of Domestic Social Robot Acceptance (de Graaf et al., 2019) presents a framework that amalgamates insights from theories of technology acceptance and research on human-robot interaction. It builds on the fundamental principles of the Technology Acceptance Model (TAM) (Davis, 1989) and the Theory of Planned Behavior (TPB) (Ajzen, 1991) and introduces unique elements of social robots. This model is characterized by its emphasis on the dual nature of attitudinal beliefs—utilitarian and hedonistic. Utilitarian attitudes consider the practical benefits of using a robot, such as efficiency and functionality, while hedonistic attitudes focus on the emotional and experiential aspects of robot interaction, such as pleasure and satisfaction. The model also emphasizes the importance of normative beliefs, which include both personal and societal norms, as well as control beliefs, which include factors such as perceived ease of use and the availability of necessary resources and infrastructure (de Graaf et al., 2019).

Three theoretical models are relevant to the adoption of domestic social robots among older adults. Firstly, the Almere Model (Heerink et al., 2010), another pivotal framework, explicitly targets the older adult population. It extends and enhances the Unified Theory of Acceptance and Use of Technology (UTAUT) (Venkatesh et al., 2003) by incorporating traditional elements such as perceived usefulness and ease of use, as well as facets of social interaction. The Almere Model is comprehensive approach that considers not only functional characteristics, but also the social dynamics that influence technology acceptance among older adults. It incorporates elements such as the perceived sociability and social presence of the technology as well as along with the user’s anxiety and perceived adaptability. This model provides an in-depth understanding of how various factors, including trust, social influence, perceived enjoyment, attitude, and facilitating conditions, jointly influence older adults’ intention to use assistive social agent technology and their actual usage behavior.

Secondly, the Model of Domestic Social Robot Acceptance (de Graaf et al., 2019) was built on the foundations of the TAM and the TPB. It focuses on three key areas: attitudinal beliefs, which include both practical (utilitarian) and pleasure-related (hedonic) attitudes toward the use of robots; normative beliefs, which emphasize the role of social influences and personal norms in the adoption of this technology; and control beliefs, which consider the perceived constraints or facilitators in the use of social robots. This model provides a framework for examining the factors that determine how domestic users perceive and interact with social robots. However, privacy and security concerns should not be overlooked when discussing the acceptance and adoption of new technologies.

Therefore, thirdly, Chatterjee et al. (2021) proposed a model that builds on the basic idea of MDSRA and additionally integrates crucial constructs such as perceived security concern, which focuses on data security and misuse of information, and perceived privacy concern, addressing fears of privacy violation. In addition, it also includes perceived legal concern, which emphasizes the legal implications of robot use. This model highlights the need to consider not only the functional aspects of domestic social robots, but also the significant privacy and security issues that affect user acceptance. By considering these aspects, the model emphasizes the importance of promoting trust and addressing security and privacy concerns, which are essential for the successful adoption and integration of these technologies into domestic environments (Chatterjee et al., 2021). Addressing privacy and security aspects is therefore crucial to advancing the field and ensuring that domestic social robots meet the needs and preferences of households.

## 2 Motivation

Despite the above-mentioned theoretical frameworks and studies on the inclusion of social robots in society (e.g., Fortunati et al., 2015; Lee et al., 2017; Horstmann and Krämer, 2019; Spatola et al., 2019), there is still a significant knowledge gap, especially regarding the acceptance of domestic social robots among older adults. Although several studies have examined social robots in the context of older adults (Lee et al., 2017; Ostrowski et al., 2019, Liu et al., 2021), there is still a shortage of studies that qualitatively address the older adults’ perceptions of domestic social robots and their security and privacy concerns. Exploring this field is essential, especially as qualitative research can provide an in-depth understanding of the ‘how’ and ‘why’ (Yin, 2014) behind older adults’ interactions and attitudes toward these robots. Such an approach is essential as older adults represent a growing population with unique needs and challenges (Zhao et al., 2022) that could be met domestic social robots could address, such as companionship (Niewiadomski et al., 2022), assistance with daily tasks (to a certain extent) (Pino et al., 2015), and support in health institutions (González-González et al., 2021) and elderly care facilities (Fraune et al., 2022).

Moreover, qualitative research allows for a deeper exploration of older adults’ concerns, particularly about privacy and security, which are of paramount importance in the context of technology adoption. By deriving our questionnaire from the model of domestic social robot acceptance (de Graaf et al., 2019) and the Almere model (Heerink et al., 2010) and including aspects of security, we aim to gain qualitative insights into these constructs that are usually measured quantitatively. Such an approach helps to understand the nuances of older adults’ perceptions and goes beyond mere acceptance to explore the emotional and psychological factors that influence their interactions with social robots.

Additionally, exploring the reasons for older people’s acceptance or resistance to the technology can provide important insights into how social robots should be designed and implemented to meet their needs and preferences. Using a qualitative approach, we can identify and address potential barriers to acceptance, such as technology-related fears, unfamiliarity with new devices or personal privacy and security concerns.

This study aims to address this knowledge gap by conducting qualitative exploratory research on older adults’ perceptions and attitudes toward domestic social robots. The study focuses on general perceptions and attitudes as well as security and privacy concerns. It aims to explore how older adults view and would interact with domestic social robots, delving into the underlying reasons and mechanisms of their attitudes. Through this exploration, the study provides comprehensive insights into the factors that should be considered when further investigating the adoption and integration of domestic social robots into the daily lives of older adults. The aim of this study is to explore the attitudes, concerns and potential adoption behavior regarding domestic social robots. Therefore, we posed the following research question:

**RQ:** How can older adults be categorized into distinct technology adoption categories based on their attitudes toward domestic social robots and their behavior in using the available technology?

## 3 Materials and methods

To answer the research question, we adopted an exploratory qualitative research design. We conducted semi-structured interviews with older adults. This section provides details on the methodology used.

### 3.1 Participants

Twenty-four retired older adults, aged 65 or older, living in the Republic of Slovenia (EU) participated in this study. The criteria for inclusion of participants in the study were: Age 65+ and ability to exercise their rights. The exclusion criterion was the presence of obvious visual or hearing impairments that could hinder the interview. Most importantly, we interviewed all older adults, regardless of their actual use of information and communication technology. Interviewing with a broader population should give us a better understanding of attitudes toward social robots. Table 1 shows the demographics of the participants.

**Table 1:** Demographic data of the participants (*n* = 24).

**Demographic parameter**	**Values**		
		**M (SD)**	**[Min., Max.]**
Age		75.6 (6.4)	[65, 87]
		**n (%)**	
Gender	Male Female	11 (45.8%) 13 (54.2%)	
Living alone	Yes No	6 (25.0%) 18 (75.0%)	
Area of living	Urban Rural	19 (79.2%) 5 (20.8%)	
Level of education	Primary or less Secondary of vocational BSc/BA or more	5 (20.8%) 16 (66.7%) 3 (12.5%)	

### 3.2 Instrument

An open-ended questionnaire served as a guide for the interviews, and additional follow-up questions were used to prompt the participants to elaborate on interesting issues that emerged during the interview. The questionnaire was designed to measure attitudes toward social robots, following the abovementioned frameworks, i.e., the Model of Domestic Social Robot Acceptance (de Graaf et al., 2019) and the Almere model (Heerink et al., 2010), while also including the construct of perceived safety and security, as suggested by Chatterjee et al. (2021), and inspired by the factors mentioned by Mihelič and Žvanut (2022). The questions were designed considering the constructs from above-mentioned literature and the specifics of the target population (e.g., short questionnaire, use of clear and concise language, avoidance of complex and excessively long questions, avoidance of similar questions where older adults would have difficulty distinguishing the answers). The questionnaire was revised for clarity by an expert in gerontology. The identified constructs and the corresponding questions are listed in Table 2. The instrument also included demographic questions and questions to determine participants’ prior knowledge of social robots, as well as a screening question to verify that participants correctly understood the possibilities of using social robots. Another important element was a short, one-minute oral information presentation using a printed image of a social robot. The aim of this presentation was to make it clear that the interviewer and the participant were talking about the same thing, namely the social robot. Special care was taken to design this presentation in such a way that it would have as little impact as possible on the participants’ responses.

**Table 2:** Identified constructs, corresponding questions and their sources.

**Construct**	**Question**	**Source**
Previous knowledge of social robots	Have you heard of social robots (i.e., robots that are part of a smart home and can assist with simple tasks)? IF YES, what do you know about them?	–
Screening question – understanding verification	Can you now imagine for what purposes such robots are used? Please provide one or two examples.	–
Use of mobile phone	Do you use a mobile phone? If yes, for which purpose you use it (using apps and internet or not)?	Mihelič and Žvanut (2022)
General attitudes	What are your general attitudes toward social robots? (Do you tend to have positive or negative feelings about using new technological devices like social robots?)	Heerink et al. (2010)
Social robot anxiety	How do you feel about the idea of using a social robot? Do you have any concerns or anxieties about talking to or using a robot in your daily life?	Heerink et al. (2010)
Utilitarian attitudes / Perceived usefulness	How useful would you find using a social robot? For example, what practical functions or tasks would you expect a robot to help you with in your daily activities?	de Graaf et al., (2019) Chatterjee et al. (2021)
Hedonic attitudes / Perceived enjoyment	Do you expect any enjoyable experiences when using a social robot? If yes, what kind of enjoyable experiences or personal satisfaction would you expect from interacting with a robot? / Do you expect to derive enjoyment or pleasure from using a social robot? How important is it for you to feel joy or satisfaction while interacting with the robot?	de Graaf et al. (2019) Chatterjee et al. (2021)
Behavioral intention	In case there would be an option for you to use a social robot, would you use it?	de Graaf et al. (2019) Heerink et al. (2010)
Perceived ease of use	Do you think using a social robot would be easy for you to use? Would you find it effortless to interact with and control the robot? Or do you anticipate any challenges or difficulties?	
Perceived safety and security using the robots	Privacy concerns can arise when using technology. Would you feel safe using a social robot? Are you worried about your personal information being exposed or any other potential risks?	Mihelič and Žvanut (2022) Chatterjee et al. (2021)
Trust	Would you trust the robot to perform the daily tasks for you? What can influence this trust?	Heerink et al. (2010)
Social influence/stigmatization	How do you think your peers and relatives would react to your use of domestic social robot?	Heerink et al. (2010)

### 3.3 Data collection

The data collection took place between 26 June and 14 September 2023. A data collection protocol was drawn up to improve the reliability of the collected data. In-depth individual interviews were conducted lasting between 20 and 30 min and were conducted by an independent interviewer, a nurse with extensive experience with this population. Special care was taken to find an interviewer who had a neutral attitude toward the use of social robots in general and specifically in this population. The interviews were conducted at a time and place that was convenient for the participant. To obtain trustworthy responses, we did not use recording devices, as the mere fact of being recorded could lead to response bias. Instead, each response was immediately noted on the interviewer’s computer. Regardless of the response to the prior knowledge question, a brief, one-minute presentation of social robots, followed by a screening question. All participants gave 1–3 plausible examples of possible uses of social robots and were all included in the rest of the study. After each interview, the transcript was reviewed with the participant, who confirmed the authenticity of the responses. The detailed protocol is presented in the Appendix. Although there are no fixed rules for sample size in qualitative research, the literature suggests that 20–30 interviews are sufficient to achieve data saturation (i.e., the point in a study at which no new relevant codes, categories or themes emerge). Initially, six interviews were conducted by randomly selecting participants. New participants were recruited through several people they trusted or through recommendations from previous interviewees. To avoid selection bias, special care was taken to select a heterogeneous sample with a variety of opinions about social robots. Data saturation was reached with the 19th interview. However, five more interviews were conducted to increase the trustworthiness and reliability of the information collected.

### 3.4 Data analysis

To improve the validity of the study, both authors conducted the coding process independently. This began with a naïve reading of the transcripts and continued with open coding, creating categories and themes according to recommendations in the literature (Braun et al., 2019). The coding process was repeated until both researchers consolidated the codes, categories, and themes, followed by the final phase in which no discrepancies were found between both researchers. The data were analyzed using Atlas.ti 7.

### 3.5 Ethical considerations

The Ethics Commission of the Faculty of Criminal Justice and Security, University of Maribor, Slovenia, EU approved this study. Participation in the study was entirely voluntary, and participants had the freedom to withdraw at any time without fear of negative consequences. Participants were invited to take part in the research following a short presentation outlining the purpose of the study and participants’ rights. In consideration of older adults’ preference to avoid signing documents for unfamiliar individuals, we decided against a written informed consent form despite initial considerations. Instead, verbal consent was obtained. Importantly, none of the participants refused to take part in the study. No additional financial support was provided for this study.

## 4 Results

**Figure 1:**
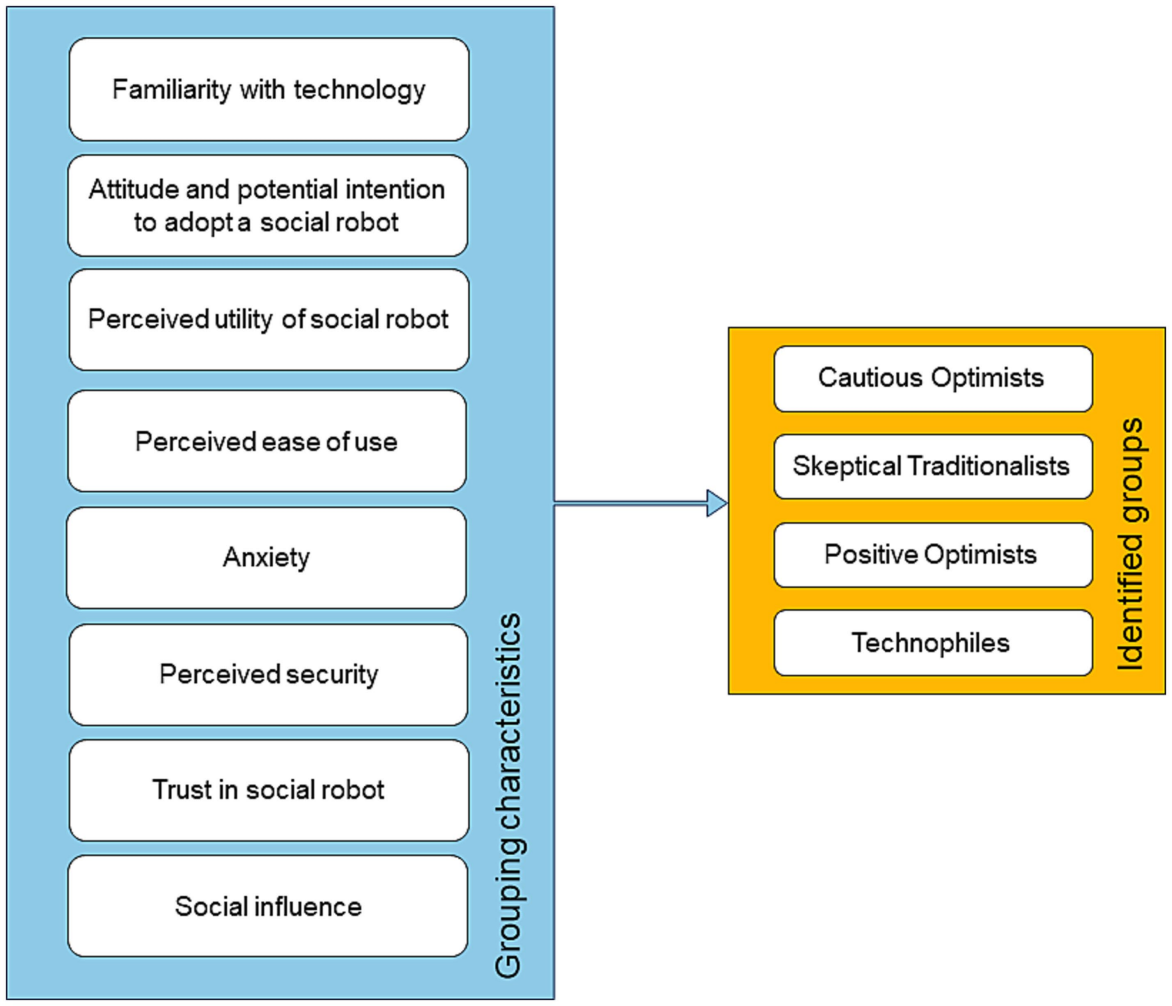
Key themes and the corresponding categories.

The results of the qualitative analysis show two main themes: (1) *Identified groups* and (2) *Grouping characteristics*. The first one consists of four distinct categories, i.e., groups of older adults who differ in their attitudes toward domestic social robots and their behavior in using the available technology. The second theme comprises eight categories, i.e., grouping characteristics (Figure 1) that served to categorize the older adults into each of the identified groups. All categories of the *Grouping characteristics* theme can be coded as positive, neutral, or negative, reflecting the connotation of each category by the participants. Eight grouping characteristics were selected from all categories identified during the data analysis. A category identified in the qualitative data analysis was considered a grouping characteristic if at least five (approx. 20%) positive or negative connotations about the characteristic were identified in the responses. Both researchers independently identified the four groups from the participants’ responses by assuming that the characteristic is typical of the corresponding group (i.e., the majority of 80% of the participants share the same characteristic connotation). In cases where responses could be assigned neither a positive nor a negative connotation, they were coded as neutral.

Table 3 summarizes the typical characteristics for each identified group. Each participant was categorized into one of the four identified groups.

**Table 3:** Summary of typical characteristics of four groups.

**Characteristic**	**Group**			
	**1** **Cautious Optimists**	**2** **Skeptical Traditionalists**	**3** **Positive Optimists**	**4** **Technophiles**
Familiarity with technology	+	−	−	+
Attitude and potential intention to adopt a social robot	+	−	+	+
Perceived utility of social robot	○	−	+	+
Perceived ease of use	+	−	−	+
Anxiety	○	○	−	+
Perceived security	−	○	○	+
Trust in social robot	○	−	+	+
Social influence	−	○	+	○

Legend: + Positive, ○ Neutral, − Negative connotation about particular characteristic.

To ensure the confidentiality and privacy of our interviewees, no specific age information was provided when reporting the results. Each quote is therefore accompanied by a unique code assigned to the interviewee (referred to P*x*, where *x* stands for a specific individual), gender, and age group. The age groups are categorized as follows: Age Group A for 65–70 years, Age Group B for 71–75 years, Age Group C for 76–80 years, Age Group D for 81–85 years, and Age Group E for 86–90 years.

### 4.1 Group 1: cautious optimists

The first group are the “Cautious Optimists” older adults. They are predominantly familiar with technology as they are typical mobile phone users and use both apps and the internet. They generally have a favorable attitude toward technological advances, particularly social robots:

“*They’re fine. It’s a gadget that can be beneficial.*”[P5, male, Age Group B].

Mainly, they are willing to adopt such innovations as they generally expect the opportunity to test and learn:

“*I have an interest in trying out every new technology to stay updated, whenever possible*.” [P9, male, Age Group A].

Nevertheless, they have significant concerns about potential privacy and security threats when using these robots, for example:

“*Yes, that can be a concern. It listens to you, and then it sends that data to stores or pharmacies. Or perhaps, when you are not home, you might get robbed.*” [P12, female, Age Group C].

Despite their overall positive attitude toward technology, they are somewhat apprehensive about the reception and reactions of their peers, leaning more toward expecting negative or neutral views:

“*At the moment, our opinion would be that it’s not necessary for me given my condition and health.*” [P24, male, Age Group B].

Cautious Optimists’ distinctive characteristic is its combination of optimism with underlying concerns. In contrast to the Technophiles group who full-fledged embrace of technology without major reservations, the Cautious Optimists exhibits caution, especially with regard to privacy. Their overall positive attitude contrasts sharply with the skepticism of the Skeptical Traditionalists. Although they recognize the potential benefits of domestic social robots in a similar way as Positive Optimists, the Cautious Optimists are distinctively more anxious about privacy and security issues.

**Typical older adult from cautious optimists group**

An older adult who uses their mobile phone not only for voice calls, but also for activities such as browsing the internet, online shopping, or using social media apps. They see the promising and potential benefits of domestic social robots and express a general positive disposition toward adopting them. However, they unanimously express concerns about the potential privacy and security issues associated with the use of this technology. When discussing the potential use of social robots with their peers, they expect mixed reactions, with some being neutral and others possibly negative. The decision to adopt such technology would be influenced by guarantees or assurances regarding security and privacy.

### 4.2 Group 2: skeptical traditionalists

The second group embodies the “Skeptical Traditionalists” among older adults. They are predominantly basic users of mobile phones, which they use for voice calls or texting. Their perspective toward technology, especially social robots, tends to be more reserved, with the majority harboring a negative attitude. The concept of social robots does not appeal to them much in terms of utility, with most perceiving limited usefulness:

*“I do not see a function for me. I have everything I need at home.”* [P6, male, Age Group C].

While they do not exhibit significant concerns about privacy and security, they remain largely not willing to use domestic social robots.

“*I’m a bit of a dumb when it comes to use of such things.*”[P10, female, Age Group C].

When it comes to social influence, this group primarily believes that their peers would take a neutral stance on the use of robot:

“*If I were to use it, I do not care about the opinions of others.*” [P19, male, Age Group B].

This group stands apart mainly due to its skepticism toward technology and robots. This cautious attitude contrasts with the optimism of Groups 1 and 4. The predominant basic phone usage also suggests that they are less familiar or comfortable with technology compared to the other groups. Their limited concerns about privacy make them unique, especially when contrasted against Group 1’s significant concerns in this domain. Another distinguishing factor is their perception of the robot’s utility, which is largely negative compared to other groups.

**Typical older adult from skeptical traditionalists group**

An older adult primarily using his/her mobile phone for essential functions such as voice calls, possibly texting or setting alarms. They are less interested in advanced features or applications. When confronted with the concept of domestic social robots, they are hesitant, mainly because they do not find them particularly useful. They are not very concerned about privacy and security, possibly because they are less familiar with these aspects or do not understand them as well. In discussions about the use of robots, they assume that most of their peers would either not have a strong opinion on it or would even see it as an unnecessary complication. The idea of integrating a robot into their daily life is not particularly appealing, and they would probably need more convincing of the tangible benefits and ease of use before considering adoption.

### 4.3 Group 3: positive optimists

The third group can be labeled as the “Positive Optimists.” They are predominantly basic mobile phone users, but they express a considerable positivity toward domestic social robots, for example:

“*As everything advances, so does technology, and I think that such a robot could come in handy*.” [P4, female, Age Group E].

Their recognition of the potential advantages of robots is evident as they all perceive a high degree of usefulness. The participants indicated various uses, e. g, “*finding telephone numbers of the physician/pharmacy, weather forecast, promotions*” [P2, male, Age Group D], “*medication reminder*” [P3, female, Age Group B]. However, a characteristic trait of this group is their mixture of emotions: While they are enthusiastic about the potential benefits, reservations about interacting with social robots may be expected in this group, for example:

“*It would be hard for me to learn how to use it. I already use my phone just for communication.*” [P3, female, Age Group B].

Their opinions on ease of use skew toward finding robots challenging, typically they are generally not very concerned about privacy.

*“If it’s designed that way, and I see that it’s effective, I would trust it.”* [P16, female, Age Group C].

When considering the views of their peers, they expect a mixture of reactions, from positive endorsements to neutral stances:

“*Well, I think they would be satisfied. They would not be against that I use it.”*” [P7, male, Age Group B].

The main distinction of Positive Optimists is their mixture of optimism and reservations. While they rate the potential utilities of robots highly, similar to Technophiles group, they differ in their concerns about interacting with social robots (anxiety) and the challenges of using. Their concerns regarding ease of use are in contrast to Groups 1 and 4, who predominantly perceive social robots as easy to use. This group is also characterized by a mixture of social influence perceptions, ranging from positive to neutral.

**Typical older adult from positive optimists group**

An older adult who uses his/her mobile phone mainly for basic tasks, but is open to technological advances and recognizes the potential of domestic social robots to improve daily life. They believe that robots could offer tangible benefits in daily activities. However, they also express reservations, feeling anxious about the idea of interacting with a robot. When considering the feasibility of using a robot, they anticipate some challenges in terms of operation and handling. They do have privacy concerns, but this is not a predominant concern for all of them. When talk to their peers about the possible use of domestic social robot, they anticipate a variety of reactions: Some peers are supportive, others are indifferent, and some may have reservations.

### 4.4 Group 4: technophiles

The fourth group epitomizes the “Technophiles” among the older adult population. They are generally typical users of mobile phones, indicating that they are familiar and comfortable with technology. They exhibit a positivity toward technological advances as well as domestic social robots as well.

“*Yes, the idea seems good to me, especially if you are alone.*” [P13, male, Age Group D].

All members believe in the substantial utility of robots and expect them to play a meaningful role in everyday tasks, e.g., for “*personal security*” [P23, female, Age Group A],” *playing* “[P14, female, Age Group A]. A notable characteristic of this group is their sheer confidence: none of them express anxieties about using or interacting with robots:

“*If I have the money for it and see that it comes in handy... why not.*” [P18, female, Age Group B].

Their perception about the domestic social robots’ ease of use is unanimous, anticipating a hassle-free experience. Even in terms of privacy and security, they remain unimpressed and feel quite safe, for example:

“*Yes, I would trust. It would not have much of an impact, as it’s designed to be trustworthy.* “[P8, male, Age Group E].

When thinking about how their peers might react, the majority expect neutral reactions, with a sprinkle of positive endorsements.

“*I take into account the opinions of others, but if it would benefit me, I would ultimately make the decision myself.*” [P21, female, Age Group A].

This group stands out primarily because of their unwavering positivity and confidence in technology. Their uniform absence of anxiety and unanimous belief in domestic social robots’ ease of use clearly sets them apart from the other groups. The predominant typical mobile phone use in this group further underscores their affinity for technology, in contrast to the basic phone users in the Skeptical Traditionalists and Positive Optimists groups. Their minimal concerns about privacy starkly oppose Group 1’s apprehensions in the same domain.

**Typical older adult from technophiles group**

An older individual who actively uses their mobile phone for a range of tasks, such as browsing the internet, using apps, and social media. They are enthusiastic proponents of technological advances and see domestic social robots as a promising extension of these possibilities. The utility of these robots for daily activities are clear to them and they expect to be instrumental in various tasks. They have no concerns or anxieties about interacting with domestic social robots and believe that using these robots is easy and intuitive. Privacy and security concerns related to domestic social robots barely register on their radar. When talking to their peers about the idea of adopting a domestic social robot, most of them are neutral stance, while a few express support and enthusiasm.

## 5 Discussion

Domestic social robots are gaining more attention across all demographic groups due to their ability to enhance daily life through automation, companionship, and assistance. For older adults, in particular, these robots offer several benefits. They can help with routine tasks, provide companionship, and alleviate the feelings of loneliness and isolation that older adults often experience. Their interaction capabilities, such as conversation and responsive behavior, can also stimulate mental engagement, which is important for the cognitive health of these people. Furthermore, thanks to technological advances, these robots are increasingly able to monitor health conditions, remind people to take their medication, and even alert emergency services if necessary, making them a valuable assistant for the well-being and even safety of older adults.

The adoption of domestic social robots among older adults, as illuminated by the results of our study, aligns intriguingly with Rogers’ Diffusion of Innovations (DOI) theory (Rogers, 2003). This theoretical framework can help us understand how different groups of older adults can be positioned on the spectrum of technology adoption, particularly in relation to their attitudes and behaviors toward social robots. The Cautious Optimists group exhibits characteristics that align them with the Early Adopters category in Rogers’ theory. Due to their familiarity with the technology and their generally positive attitude toward domestic social robots, they are potentially open to adopting such innovations. However, their concerns about privacy and security, as well as their caution about peer perception, suggest that they are hesitant to be the first to adopt this technology. This hesitation sets them apart from the Innovators group, who are generally more willing to take risks and be the first to embrace new technologies (such as Technophiles).

The Technophiles group embodies the characteristics of innovators or early adopters. Their enthusiasm for technology, their belief in the significant utility of social robots, and absence of anxiety about using them are consistent with the traits of Innovators. They are most likely to adopt social robots quickly because they have confidence in the technology and have little concern about privacy and security. Their high level of tech-savviness and proactive attitudes toward technological advancements make them potential leaders in the adoption of social robots among older adults.

The Skeptical Traditionalists group best reflects the Laggards or, at best, the Late Majority. Their basic use of mobile phones for primary functions and their general skepticism about domestic social robots indicate a strong resistance to adoption. This group’s view that domestic social robots have limited utility and their general disinterest in technological advances may even categorize them as Non-adopters. They pose a major challenge to the diffusion of social robots as they are less likely to be influenced by trends or peer pressure and adhere instead to traditional values and views.

The Positive Optimists group aligns with the late majority. This group shows a cautious yet positive attitude toward domestic social robots. Notable reservations about the ease of use and interaction with these robots reinforce their recognition of the potential benefits. This cautious optimism is symbolic of the late majority, who are generally slower to adopt new technologies. This cautious of this group suggests that they are reluctant to adopt new technologies quickly, which aligns them more closely with the Late Majority, who only adopt innovations once their effectiveness and practicality have been confirmed by earlier adopters.

The adoption of social robots among older adults in the context of DOI theory reveals a diverse spectrum of technology acceptance that challenges stereotypes about older adults’ aversion to new technologies. The categorization presented in this study shows that older adults are not monolithically resistant to technological innovations. Instead, they are equally represented in categories typically associated with openness to innovation, such as innovators and early adopters, just like other demographic groups. This nuanced understanding highlights the importance of tailoring domestic social robot adoption strategies that consider the specific needs and attitudes of each subgroup. Recognizing diversity in technology adoption among older adults is critical to the effective integration of new emerging technologies and demonstrates their role as active participants in the evolving technological landscape.

In our study, privacy and security concerns emerged as pivotal factors, particularly among the Cautious Optimists and Technophiles groups. These concerns indicate an apprehension about the potential malicious data collection, misuse, or mishandling of personal data by these technological devices, which are equipped with numerous sensors, cameras, and microphones. Participants expressed concern about the domestic social robots’ ability to “eavesdrop” and potentially share sensitive information with third parties, highlighting the fear of loss of privacy and increased vulnerability. This anxiety is heightened by the realization that these robots, being connected devices, could be vulnerable to hacking or unauthorized access, posing a significant risk to the user’s personal data.

The factors underlying these concerns are diverse. One critical aspect is the varying degrees of familiarity older adults have with technology among older adults, which has a significant impact on their perception of risk (Mihelič and Žvanut, 2022). Our study indicates that those who are more experienced in using technology, such as regular mobile phone users, tend to be more aware of the privacy and security risks associated with internet-connected devices. This awareness leads to a cautious approach toward new technologies such as domestic social robots, whose potential benefits are recognized but which also carry risks. However, we can observe that very high levels of familiarity with technology (i.e., Technophiles) can also lead to greater confidence when it comes to tackling security issues themselves; thus, higher awareness may not always be associated with greater caution. Instead, it should be considered in a broader context, such as other factors identified in our study.

Additionally, the level of trust in the manufacturers of these robots plays a crucial role. Concerns about how data is collected, stored, and used and uncertainties about who has access to this data contribute to a reluctance to fully trust these devices. The impact of these privacy and security concerns on the adoption of domestic social robots among older adults is noteworthy. They act as a barrier and overshadow the recognized benefits of these technologies. For many, fear of privacy and security breaches is a significant barrier, preventing them from adopting these technologies. Therefore, reassurance and robust measures are needed to ensure the security and privacy of user data. Understanding and mitigating these concerns is vital for the wider acceptance and integration of social robots into the lives of older adults.

Our findings reveal a complex interplay between perceived utility and ease of use, influenced by their existing technology use and behaviors. The perceived utility of domestic social robots among older adults varies significantly. While groups such as the Technophiles and the Positive Optimists recognize the potential benefits of these robots for improving daily life, their enthusiasm is contrasted by the skepticism of the Skeptical Traditionalists, who see limited utility. The Cautious Optimists, who are typically familiar with the technology, appreciate the potential benefits but are held back by privacy and security concerns. This variation in perceived utility reflects the varying levels of engagement with the technology across groups and the resulting expectations of robotic assistance in their daily lives.

The perceptions of the ease of use of domestic social robots also display considerable variation. Technophiles, who are confident in their technical abilities, expect a straightforward and hassle-free experience with these robots. In contrast, the Positive Optimists express concerns about the ease of interaction and learning, although they recognize the potential benefits of domestic social robots. Their concerns point to a need for user-friendly design and clear instructions are needed to facilitate adoption. With their limited technology use, Skeptical Traditionalists doubt their ability to grasp the complexity of robotic technology. This suggests a gap between the capabilities of the technology and the comfort level of users. These findings highlight the importance of considering the different technological backgrounds and attitudes of older adults when introducing domestic social robots.

The role of social influence in the adoption of domestic social robots by older adults is characterized by complex and multifaceted dynamics. This aspect becomes particularly interesting when we consider the neutral connotation of social influence for certain groups, such as the Skeptical Traditionalists and the Technophiles, as opposed to its significant role highlighted in prior research like Heerink et al. (2010) and Chatterjee et al. (2021). Initially, it is important to recognize how differently social influence is pronounced across different groups of older adults. The Cautious Optimists and Positive Optimists may be more susceptible to social norms and peer opinion, which is consistent with the findings of Heerink et al. (2010) that social influence significantly affects attitudes toward technology. This influence could be due to their balanced view of technology – they are neither fully resistant nor fully embracing, which makes them more receptive to external opinions.

In contrast, the Skeptical Traditionalists and the Technophiles demonstrate a notable departure from this pattern. Their neutral stance toward social influence indicates a more autonomous decision-making process about the adoption of social robots. For the Skeptical Traditionalists, this autonomy could be due to skepticism or disinterest in the technology, making them less likely to be influenced by the opinions of others. They seem to base their decisions more on personal experience and perceived utility rather than social norms or peer pressure. Technophiles, on the other hand, show confidence and a positive attitude toward technology, which could make external social influences less significant in their decision-making process. Their enthusiasm and familiarity with technology suggests that their adoption choices are driven by personal interests and perceived benefits rather than the opinions or behaviors of their peers.

Our study provides a detailed and nuanced understanding of older adults’ attitudes and perceptions toward domestic social robots emerging from qualitative research. The qualitative approach confirmed that several factors proposed in the Almere model (Heerink et al., 2010) e.g., trust and anxiety, may be more relevant to older adults than originally thought and confirmed that factors, such as usefulness, ease of use, and social influence are indeed pertinent to categorize older adults into specific adoption groups. Furthermore, factors such as anxiety and trust could be directly related to older adults’ desire to own or not own a domestic social robot.

On the spectrum of utilitarian and hedonic attitudes, as suggested by de Graaf et al. (2019) and Chatterjee et al. (2021), we found that such attitudes (often understood interchangeably by older adults) are a decisive factor for categorization (either by positive or negative attitudes) in three out of four groups. Meanwhile, the perceived security issues emphasized by Chatterjee et al. (2021) are more polarizing and can be used as a distinctive characteristic seen as an issue only by skeptics. Social norms and personal norms, as postulated by de Graaf et al. (2019), are shown in our research only as social influences, trust, and privacy concerns as distinct factors (and not as an integrated construct of “personal norms”). This suggests that a more detailed approach is needed when developing predictive models.

These variations in the impact of social influence emphasize the need for nuanced models for understanding technology adoption among older adults. Traditional and general models cannot fully capture the complexity of this population, particularly regarding the influence of social factors. The neutral connotations observed in our study suggest that predictors of technology adoption that are significant in general populations may not carry the same weight in all groups of older adults.

### 5.1 Implications

This study yields several theoretical and practical implications. First, to our knowledge, this is the first study to propose a categorization of older adults based on established frameworks and qualitative research that captures the nuanced spectrum of attitudes and behaviors toward technology in this population. By integrating insights from established models such as the Model of Domestic Social Robot Acceptance (de Graaf et al., 2019) and the Almere model (Heerink et al., 2010) with qualitative data, the study provides an approach to understanding how older adults would interact with and perceive new technologies such as domestic social robots. This categorization can serve as a valuable theoretical tool for future research in technology adoption among older populations. The identified themes and categories shown in Figure 1 are indeed a starting point for the development of the theoretical model. This requires an appropriate and careful transformation of the themes and categories into quantitative variables, the development or adaptation of valid and reliable data collection instruments, the collection of representative data, and finally the testing of the predictive power of the model using appropriate statistical methods.

Second, this study challenges the existing notion of social influence in technology adoption, emphasizing that its impact is not the same for all groups. The findings reveal that the effect of social influence on the adoption of social robots among older adults varies considerably, depending on which group they belong to. This nuanced view encourages a re-evaluating of the way social influence is conceptualized and integrated into models of technology adoption among older adults.

Third, the emphasis on privacy and security concerns among older adults in this study indicates that theoretical models need to incorporate these aspects more explicitly. It is important to understand how these concerns interact with other factors identified in this study. This integration may provide a more comprehensive framework for predicting technology adoption behaviors in older adults.

Fourth, designers and developers should customize domestic social robots to meet the specific preferences and needs of identified groups among older adults. A user-friendly interface, built-in rigorous privacy settings, and robust security measures can help address the various concerns and increase adoption.

Fifth, the study suggests the importance of tailored educational initiatives for older adults to promote familiarity and competence with technology. These initiatives, including practical workshops and training, are crucial in addressing the specific concerns and characteristics identified in the research. For example, addressing perceived privacy and security issues in the Cautious Optimists group and building trust in social robots in the Skeptical Traditionalists group. These educational efforts are important not only to encourage older adults to purchase these technologies, but more importantly, to help them make informed decisions. In this way, these initiatives help to close the knowledge gap and reduce uncertainty, enabling a more confident and informed use of technology.

Sixth, the role of social influence, particularly in Cautious Optimists and Positive Optimists groups, emphasize the need to create and promote peer engagement and support groups. These platforms can enable older adults to share their experiences and testimonials about social robots, fostering a collaborative learning and support system. Such peer-to-peer interactions can have a substantial impact on older adults’ perceptions and adoption decisions. By observing and discussing real-life applications and benefits of social robots with their peers, older adults may become more open and receptive to adopting these technologies. This approach harnesses the power of social influence and allows for a more organic and credible form of persuasion than traditional marketing strategies.

### 5.2 Limitations and future work

This study has several limitations that should be noted. First, the study included older adults from one country, which limits the generalizability of our findings. Therefore, further studies should be conducted in other sociocultural contexts to increase the external validity of our results and to gain a comprehensive understanding of older adults’ attitudes toward domestic social robots. Second, the use of interviews may limit the trustworthiness of participants’ responses due to social desirability or even intentional vagueness of responses. Third, the absence of recording devices during the interviews, while intended to ensure participant comfort, may have led to the potential loss of data richness or nuance in responses. Fourth, the recruitment method based on a combination of random selection and recommendations from previous interviewees, while beneficial in avoiding selection bias, may not fully represent the diversity range of older adults. There is a potential for homogeneity in opinions or backgrounds, especially if the recommended participants share similar views or social circles. Fifth, a notable limitation of the study is that participants did not own or regularly use a domestic social robot. This lack of direct experience with the technology may influence their perceptions and responses. Participants’ attitudes and opinions were based on hypothetical scenarios or their understanding of social robots rather than actual usage experiences. This could lead to a gap between the attitudes expressed attitudes and the possible behaviors or preferences in the real world if they were to interact with these robots on a regular basis.

Future research should primarily involve the quantitative validation of the categorization of older adults’ attitudes toward domestic social robots. This could involve designing a survey that captures a broader demographic and allows for statistical analysis of the identified categories. In addition, the use of classification techniques could further validate and refine this categorization, allowing for a more nuanced understanding of older adults’ views on technology. Second, given the geographical limitation of the study to Slovenia, it would be beneficial to conduct similar research in other countries. Comparative studies in different cultural, economic, and social settings may shed light on the extent to which these findings are culturally specific or universally applicable. Third, investigating the impact of educational initiatives tailored to older adults in the context of social robots is another potential area for future research. Evaluating the effectiveness of different forms of training and educational programs can provide insights into how best to promote older adults’ understanding, acceptance, and adoption of these technologies. Finally, future research should also include participants who have had direct experience with domestic social robots, as this could provide more informed insights into usage patterns, benefits, and challenges, and thus providing another aspect of understanding the acceptance and adoption process among older adults.

## Data availability statement

The raw data supporting the conclusions of this article will be made available by the authors, without undue reservation.

## Ethics statement

The studies involving humans were approved by Ethics Commission of the Faculty of Criminal Justice and Security, University of Maribor. The studies were conducted in accordance with the local legislation and institutional requirements. Written informed consent for participation was not required from the participants or the participants' legal guardians/next of kin because the participants were invited to participate in the research following a brief presentation outlining the study's purpose and participants' rights. In consideration of older adults' preference to avoid signing documents for unfamiliar individuals, we chose not to implement written informed consent, despite initial consideration. Instead, consent was obtained orally.

## Author contributions

BŽ: Conceptualization, Data curation, Formal analysis, Investigation, Methodology, Software, Visualization, Writing – original draft, Writing – review & editing, Funding acquisition. AM: Conceptualization, Data curation, Formal analysis, Investigation, Methodology, Resources, Supervision, Visualization, Writing – original draft, Writing – review & editing.
