# Supplementary material for: Qualitative study on domestic social robot adoption and associated security concerns among older adults in Slovenia
Source: Front Psychol. 2024 Jan 25;15:1343077. doi: 10.3389/fpsyg.2024.1343077 (PMC10850379; doi:10.3389/fpsyg.2024.1343077)
Supplement: Supplementary file 1 [file Table_1.DOCX]

Qualitative Study on Domestic Social Robot Adoption and Associated Security Concerns Among Older Adults in Slovenia

Boštjan Žvanut^1^†, Anže Mihelič^2^† ^*^

*** Correspondence:** Anže Mihelič anze.mihelic(at)um.si

† These authors contributed equally to this work and share first authorship.

**Appendix: The interview protocol**

1. Begin by introducing yourself and stating the institutions you represent.
2. Briefly explain the study's objective, which is to explore attitudes, concerns, and potential behaviors regarding the adoption of domestic social robots. Outline the interview process, emphasizing its duration of 20-30 minutes and the immediate recording of responses on the interviewer’s computer.
3. Assure the participant of their anonymity and the voluntary nature of their participation. Inform them that they can withdraw from the interview at any time.
4. Seek the participant's consent by asking, "Do you agree to participate in this interview?"
5. If the participant consents, proceed to step 6. If not, politely conclude the interview, assuring them that it is perfectly acceptable.
6. Collect the participant's demographic data (gender, area of residence - urban or rural, age, living status - alone or not, and education level) and input it into the designated sections of the form.
7. Inquire about the participant’s prior knowledge of social robots. Regardless of their response, spend a minute explaining what a social robot is, using a neutral tone, and show a provided photo.
8. Pose a screening question: "What do you think are the potential applications of social robots? Please provide 1-3 examples." Continue the interview if the responses are reasonable. Terminate the interview only if all responses are nonsensical.
9. Proceed with the questions listed in Table 2.
10. Encourage the participant to elaborate or clarify their responses if needed.
11. Record each response in the corresponding section of the form on your computer.
12. After the interview, review the transcript with the participant and ask for confirmation of the accuracy of their responses. Record their confirmation in the form.
13. Express gratitude to the participant for their cooperation and provide our contact information for any further queries they might have.
